# From resistance to persistence: Insights of a mathematical model on the indiscriminate use of insecticide

**DOI:** 10.1371/journal.pntd.0008862

**Published:** 2020-11-18

**Authors:** Helio Schechtman, Denise Valle, Max O. Souza

**Affiliations:** 1 Programa de Computação Científica, Fundação Oswaldo Cruz, Rio de Janeiro, Brazil; 2 Laboratório de Biologia Molecular de Flavivírus, Insituto Oswaldo Cruz, Fundação Oswaldo Cruz, Rio de Janeiro, Brazil; 3 Instituto Nacional de Ciência e Tecnologia em Entomologia Molecular (INCT-EM)/CNPq, Rio de Janeiro, Brazil; 4 Instituto de Matemática e Estatística, Universidade Federal Fluminense, Niterói, Rio de Janeiro, Brazil; Institute for Disease Modeling, UNITED STATES

## Abstract

The development of insecticide resistance is becoming a threat to many arboviruses control programs worldwide. While this has been attributed to the indiscriminate use of insecticide, a more theoretical study is apparently not available. Using *in-silico* experiments, we investigated the effects of two different policies: one used by the Brazilian Ministry of Health (which follows the World Health Organization protocol) and a more permissive one, akin to those employed by various gated communities and private companies. The results show that the public policy does not lead to resistance fixation. On the other hand, permissive application of adulticide, such as intensive domestic use mainly during epidemic periods, might lead to the fixation of a resistant population, even when resistance is associated with moderate fitness costs.

## Author summary

Mosquitoes populations are typically very large, and the fraction of insecticide resistant individuals in these populations has been observed to be increasing in recent field measurements. This is commonly attributed to the indiscriminate use of insecticides, but it also suggests that small fitness costs might also come into play.

Using a mathematical model for the mosquito population, we study the impact of different application policies on the development of resistance in a population that was initially almost completely susceptible. We focus on two different policies: one that can be associated with a more restrained public policy, and a more permissive that is typically found in gated communities.

The results suggest that a more permissive use not only increases the fraction of resistant individuals in the population, but might also lead to the replacement of a susceptible population by a resistant one. They also show that, while such a permissive policy initially brings down the overall mosquito population, the development of resistance claims its price and it diminishes the efficacy of insecticide control in the long term scenario.

While this is perhaps an oversimplified model to be used quantitatively, it captures enough of the qualitative underlying biological and ecological features, and foster further discussions and studies on this issue.

## Introduction

Currently, the threat posed by *Aedes aegypti* is increasing worldwide. This mosquito, already recognized as the main vector of urban yellow fever and dengue, has also been associated to outbreaks of other arboviruses infections. These include chikungunya and Zika which are spreading throughout several countries [1]. In particular, Zika has been confirmed as the cause of microcephaly in foetuses, among other neurological complications [2]. In Brazil, for instance, the dengue virus is present uninterruptedly since 1986 and its four serotypes circulate in the country since 2010 [3]. The chikungunya virus was introduced in 2014 and soon after, the Zika virus [4–6].

There are no specific treatments for the clinical manifestations of dengue, chikungunya and Zika virus nor effective vaccines available for large scale use. This situation strongly contributes to collective fear, and the search for vector control solutions is accelerated as the reduction of infection risk relies solely on control of *A. aegypti* infestation rates. Elimination of vector populations is usually almost exclusively assigned to chemical insecticides, deemed quick and effective. Perception of chemical control as the most effective tool is even more exacerbated during epidemic periods or when new diseases emerge, as currently seen in different localities [7–9]. Such a scenario results in an uncontrolled increase in insecticide applications exacerbating the conceptual confusion between “vector control” and “chemical vector control” or, more specifically, “chemical control of adult vectors” [10].

Control of *A. aegypti* populations ideally consists on the elimination of breeding sites, a practice known as mechanical control, in conjunction with judicious insecticide applications. However, the effectiveness of chemical insecticides have been jeopardized due to the spread of resistance in vector populations [11]. Domestic insecticide applications in private households or gated community areas may have contributed significantly to the observed fast deterioration of their effectiveness [10]. These gated communities are typically high-middle class located in less densely populated areas. In Brazil, such communities have private administration and security arrangements.

Insecticide resistance is genetically based and, in general, does not bestow additional advantages. For the classical neurotoxic insecticides, it is common that resistance compromises mosquito fitness. This is due to the fact that the main resistance mechanisms involve deviations of metabolic resources or some degree of impairment of specific functions of the nervous system [10, 12–15]. The former mechanism corresponds to an increase in the insect detoxification capacity, known as metabolic resistance, whilst the latter involves potential changes in the specificity or activity of target pesticide molecules [10, 16]. Insecticide resistance is usually transmitted in a recessive inheritance mode and it is silently maintained in field populations by heterozygous individuals [17]. Therefore, usually, naturally resistant specimens are at a low prevalence frequency in vector populations not subjected to chemical pressure.

Coordination of dengue vector control in Brazil has always been a responsibility of the Ministry of Health (MoH). Since 2002 the National Program for Dengue Control (PNCD in Portuguese) coordinates the *A. aegypti* surveillance and control actions [18]. Although the importance of sanitation actions and of popular mobilization towards an effective control is increasingly recognized, the PNCD still has a strong bias favoring chemical control [19]. Despite the strong technical emphasis to solve a problem that is essentially political, structural, and related to behavior, from a biomedical point of view, the *A. aegypti* chemical control in Brazil has international sustenance [20]. The Brazilian PNCD strictly follows the actions recommended by World Health Organization (WHO) for insecticide applications and only those insecticides recommended by the WHO Pesticide Evaluation Scheme (WHOPES) are employed by the MoH [21, 22].

The officially recommended protocol for chemical control of the vector *A. aegypti* is as follows. Containers that cannot be discarded and may become infested with larvae are to be treated four to six times a year by public agents when conducting inspections of houses in the communities. Control of adult mosquitoes must only be conducted to block outbreaks, and never preventively. Whenever necessary, a maximum of five to seven space spraying applications, every three to five days, are performed yearly in a given locality using motor vehicles [19].

Regarding chemical control, the official policy in Brazil only prescribes larvicides that are approved for use in potable water [22]. The organophosphate temephos was the sole compound available for this purpose for many years [23]. However, since 2009, Brazil employs different classes of Insect Growth Regulator (IGR) larvicides which are to be used in a rotation scheme, every four years, for each class [24]. Adult control was achieved, since 2000 and until recently, by the use of pyrethroids. It is worth mentioning that IGRs, in general, are not purchasable in the retail market, whilst pyrethroids may be easily acquired by the population. This certainly contributed to the rapid increase in pyrethroid resistance levels [25, 26], a situation that induced the MoH to recommend interruption of this class of adulticides [27].

Summers, which may coincide with epidemic periods, are also the season when individuals realize that initiatives against the mosquito vectors must be taken. Such private initiatives are generally restricted to the control of adult mosquitoes and are strictly based on the use of insecticides, usually pyrethroids. It is quite common, in large Brazilian cities, that gated communities hire private suppliers to conduct space spraying of insecticides. Such a procedure allegedly may be executed up to twice a day, up to five days a week.

It has been observed that space spraying may reduce the size of the vector population, more so when intensively performed as in the gated communities. However, in the medium term it might result in selection of resistant individuals, which in turn may even culminate with the irreversible fixation of this resistance characteristic in the vector population [8].

The objective of the present study was to examine and evaluate the effect of different strategies of chemical control of adult vectors by pyrethroids on the spread of resistance: the conservative procedure adopted by the Brazilian MoH, which is in accordance with the WHO recommendations, and a most extreme application, such as the one depicted above for gated communities during epidemic situations in major urban centres of the country. In what follows, the former strategy will be denoted by PUBL, whereas the latter will be denoted by PRIV. This is in agreement with the actors responsible for the major use of each strategy—PUBL(ic) or PRIV(ate).

## Materials and methods

### Biology of resistance in *Aedes aegypti*

In *A. aegypti* field populations, resistance to pyrethroids is mainly associated with a single gene, its target site, in the mosquito’s central nervous system [9, 26, 28, 29], the voltage regulated sodium channel (Na_V_). Therefore, we chose to work with the single-factor resistance model: SS, SR and RR. As with Na_V_ in nature, we consider that resistance is recessive, i.e., it is only expressed in homozygosity (RR); that is, in this case hybrids (SR) have the same phenotype as susceptible individuals (SS). We use the same rule for impact on fitness: hybrid individuals (SR) are as ‘viable’ as susceptible individuals (SS).

Insecticide resistance usually comes with associated fitness costs. Under intensive selective pressure, however, compensatory genes can be selected and decrease this cost—cf. [30]. The actual cost impact is a matter of debate: recent works have suggested that this cost can be indeed high [31], whereas assessment of field populations indicates that resistant strains are overtaking susceptible ones [32] suggesting otherwise—see also [33]. This uncertainty extends to how these costs are distributed over the various fitness components—eg. oviposition rates, death rates. In order to cope with such different views and uncertainties, and also to allow a worst scenario analysis, we chose a range of fitness costs that is biased to smaller costs. Namely, we work the set of costs given by $C_{\text{values}}=\left\{0.005,0.01,0.05,0.1,0.15\right\}$. These costs were uniformly applied at birth and death rates.

### Insecticide characteristics

In general insecticides target the larval (larvicides) or adult (adulticides) stages. Nowadays, the mainly employed larvicides are organophosphate compounds, eg. temephos, that target the central nervous system, growth regulators, eg. methoprene, or biological control agents as Bti. Adulticides are usually pyrethroids or organophosphate compounds.

The actual persistency of insecticides varies according to different factors. Presently available organophosphate larvicides can be active from one to five months, and are typically highly effective since they are applied directly in the mosquito breeding sites—we assumed a two month activity period with an efficacy of 90% based on choices made by previous studies [34–36]. On the other hand, adulticides used in space spraying applications have a very short active range (typically a few hours) and there is a lack of agreement in the literature on their efficacy: some studies suggest an efficacy of 20 to 30% [35], whereas some agricultural studies suggest much smaller values (see [37]). We opted to take a more conservative view and use the following set of efficacies: $K_{\text{values}}=\left\{1\%,6\%,10\%\right\}$.

### Mathematical model

**Fig 1:**
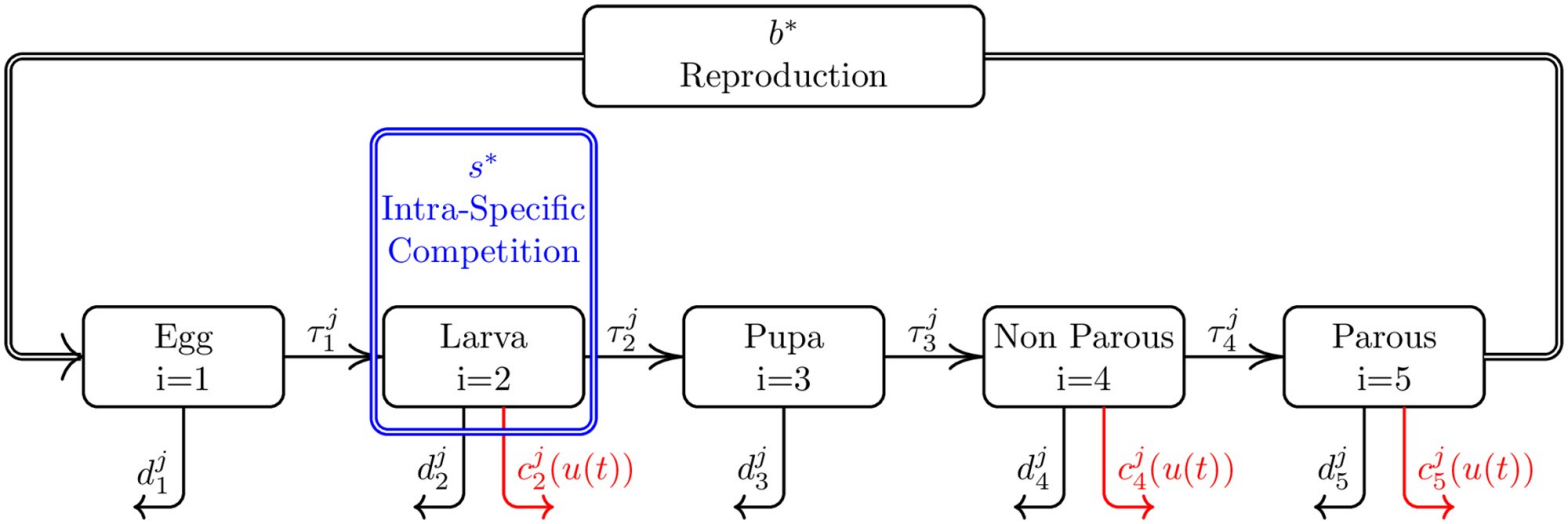
Compartmental description. The model comprises five life-stages of three mosquito genotypes that are represented by rectangular boxes. The genotypes are indexed by *j* as follows: {SSj=1,SRj=2,RRj=3}. Arrows connecting two boxes represent mosquitoes of a specific life stage maturing into another life stage, with the appropriate transition rate. Outward arrows represent removal of individuals from that specific life stage due to natural or insecticide-caused death. The thicker arrow connecting the Parous stage to the Egg stage boxes represents an influx of eggs arising from random, non-preferential mating of parous mosquitoes. The Intra-Specific Competition box represents a logistic type competition among larvae of the three genotypes. Note that interaction among different genotypes occur only at the mating and larval stages.

We used the population model described in [38], slightly modified to accommodate for insecticide application—see also [35]. The model is stage structured and assumes that resistance is mediated by two alleles, Susceptible and Resistant, in a single locus following Mendelian inheritance. As observed in Biology of resistance in *Aedes aegypti*, the single-factor resistance is modelled as being recessive. The impact on fitness was also assumed to be recessive occurring only in the resistant individuals. The model is deterministic with dynamics governed by the system of ordinary differential equations given in Eq (1) and supplemented by Eqs (2)–(11), where we write *Y*_*i*,*j*_ to denote the number of individuals at stage *i* with genotype *j*—see Fig 1 for the compartmental description and further information on the labelling of stages and genotypes.

$$\begin{array}{c} \left\{ \begin{array}{cc} {\dot{Y}}_{1,j} & =B\left(j,Y_{5,\cdot }\right)-(\tau_{1}\left(t\right)+d_{1}^{j})Y_{1,j}\\ {\dot{Y}}_{2,j} & =\tau_{1}\left(t\right)Y_{1,j}-(\tau_{2}\left(t\right)+d_{2}^{j}+c_{2}\left(j,t,u_{L}\left(t\right)\right)+s^{j}\sum_{l=1}^{3}Y_{2,l})Y_{2,j}\\ {\dot{Y}}_{3,j} & =\tau_{2}\left(t\right)Y_{2,j}-(\tau_{3}\left(t\right)+d_{3}^{j})Y_{3,j}\\ {\dot{Y}}_{4,j} & =\tau_{3}\left(t\right)Y_{3,j}-(\tau_{4}+d_{4}^{j}+c_{4}\left(j,t,u_{A}\left(t\right)\right))Y_{4,j}\\ {\dot{Y}}_{5,j} & =\tau_{4}Y_{4,j}-(d_{5}^{j}+c_{5}\left(j,t,u_{A}\left(t\right)\right))Y_{5,j} \end{array}\right. \end{array}$$ (1)

#### Oviposition function

Oviposition is described by the Mendelian mating function *B*(*j*, *Y*_5,⋅_) given by the equations below:

$$\begin{array}{c} B(1,Y_{5,\cdot })=\frac{Y_{5,1}^{2}b^{\left(1,1\right)}+Y_{5,1}Y_{5,2}b^{\left(1,2\right)}+0.25Y_{5,2}^{2}b^{\left(2,2\right)}}{Y_{5,1}+Y_{5,2}+Y_{5,3}}, \end{array}$$ (2)

$$\begin{array}{c} B(2,Y_{5,\cdot })=\frac{Y_{5,1}Y_{5,2}b^{\left(1,2\right)}+0.5Y_{5,2}^{2}b^{\left(2,2\right)}+Y_{5,2}Y_{5,3}b^{\left(2,3\right)}+2Y_{5,1}Y_{5,3}b^{\left(1,3\right)}}{Y_{5,1}+Y_{5,2}+Y_{5,3}}, \end{array}$$ (3)

$$\begin{array}{c} B(3,Y_{5,\cdot })=\frac{Y_{5,3}^{2}b^{\left(3,1\right)}+Y_{5,3}Y_{5,2}b^{\left(3,2\right)}+0.25Y_{5,2}^{2}b^{\left(2,2\right)}}{Y_{5,1}+Y_{5,2}+Y_{5,3}}; \end{array}$$ (4)

with

$$\begin{array}{c} b^{\left(j,j\right)}=b^{\left(1,1\right)}\left(1-C(j,b^{\left(1,1\right)}\right)),\;j=2,3; \end{array}$$ (5)

and

$$\begin{array}{c} b^{\left(m,n\right)}=\sqrt{b^{\left(m,m\right)}b^{\left(n,n\right)}},\;m,n=1,\ldots ,3,\;m\neq n. \end{array}$$ (6)

Eq (5) models the cost for eggs with parents that have the same genotype. For crossgenotype breeding, Eq (6) postulates an average cost given by the geometric mean—which is natural in the context of multiplicative costs assumed in the model. For further discussion see [38].

#### Other model coefficients

The remaining coefficients in Eq (1) are as follows:

$$\begin{array}{c} \tau_{i}\left(t\right)={\bar{\tau }}_{i}(1+\delta \sin(\frac{2\pi t}{\bar{y}}+\theta_{i})),\;i=1,2,3. \end{array}$$ (7)

$$\begin{array}{c} s^{j}=s^{1}(1+C(j,s^{1})),\;i=1,\ldots ,4;\;j=1,2,3. \end{array}$$ (8)

and

$$\begin{array}{c} d_{i}^{j}=d_{i}^{1}(1+C(j,d_{i}^{1})),\;i=1,\ldots ,5;\;j=1,2,3. \end{array}$$ (9)

$$\begin{array}{c} c_{2}\left(j,t,u\right)=K_{L}\Phi_{L}(\frac{t-u}{T_{L}}). \end{array}$$ (10)

$$\begin{array}{c} c_{4}\left(j,t,u\right)=c_{5}\left(j,t,u\right)=\{\begin{array}{cc} K_{A}\Phi_{A}(\frac{t-u}{T_{A}}), & j=1,2;\\ 0, & j=3. \end{array} \end{array}$$ (11)

In the above $K_{*}$, with * = *A*, *L*, denotes the adulticide and larvicide, respectively, efficacy; T_*_ is the corresponding activity time and Φ_*_(*t*) is a non-increasing function, with compact support in [0, 1], that modulates the decay of insecticide activity. The control function **u** specifies the application time. Since costs are assumed to impinge only on the resistant phenotype, we have that C(j,⋅)=0 for *j* = 1, 2.

This model has been parametrised for the biology of A. aegypti and for the climate of the city of Rio de Janeiro—see Table 1 for the parameter values used; see also [38] and references therein for further details. In summary, climate was assumed to produce a periodic fluctuation in the transition rates from one stage to another of the A. aegypti population—the transition accelerates up in the summer and slows down in the winter. Resistance costs, which were parametrized by a common intensity level, were assumed to impinge on the oviposition and natural death rates of the resistant homozygous form. Specifically, the number of eggs that are spawned is calculated as the geometric mean of the oviposition rates of the parental phenotypes. See S1 Text for details on the model initial conditions and computational implementation.

**Table 1:** Model parameters.

Parameter	Value	Definition
$d_{1}^{1}$	0.01005	baseline egg death rate
${\bar{\tau }}_{1}$	1/120	egg to larva mean transition rate
$d_{2}^{1}$	0.10536	baseline larva death rate
${\bar{\tau }}_{2}$	1/12	larva to pupa mean transition rate
$d_{3}^{1}$	0.01005	baseline pupa death rate
${\bar{\tau }}_{3}$	1/2	pupa to non-parous (winged) mosquito mean transition rate
$d_{4}^{1}$	0.02020	baseline non-parous (winged) mosquito death rate
${\bar{\tau }}_{4}$	1/5	non-parous to parous (winged) mosquito transition rate
$d_{5}^{1}$	0.06187	baseline parous (winged) mosquito death rate
*b*^(1, 1)^	4	baseline oviposition rate
*s*^1^	10^−6^	baseline per capita intra-specific death rate
*θ*_1_	1.8326	phase shift for egg to larva transition rate
*θ*_2_	1.0472	phase shift for larva to pupa transition rate
*θ*_3_	1.0472	phase shift for pupa to non-parous (winged) mosquito transition rate
*δ*	0.25	amplitude of periodic perturbation

Transition, death, oviposition rates, amplitude of periodic perturbation and phase shifts for susceptible individuals in the model depicted in Fig 1. Rates are given in *day*^−1^, the *s*^1^ parameter is given in *day*^−1^⋅*individual*^−1^ whilst phase shifts are presented in radians. The parameter *δ* is dimensionless.

The functions *u*_*A*_ and *u*_*L*_ describe the externally imposed populational control, i.e. insecticide application. The function *c*_*i*_(*u*(*t*)) describes the additional death rate due to these applications over the time span of the respective insecticide activity. The adulticide activity was taken to be constant over some chosen duration (typically a fraction of a day) whilst for the larvicide the activity was taken as lasting for 60 days with a parabolic decay over this period (see below).

### The populational control policy

The function **u**_*A*_(*t*) specifies the control policy for adulticide application. We considered two annual policies: the application policy prescribed by the Brazilian MoH, denoted as PUBL, and a far more permissive policy denoted as PRIV.

Larvicide was used in conjunction with both adulticide applications. The control policy for larvicide application **u**_*L*_(*t*) was the same independently of the adulticide policy.

**Fig 2:**
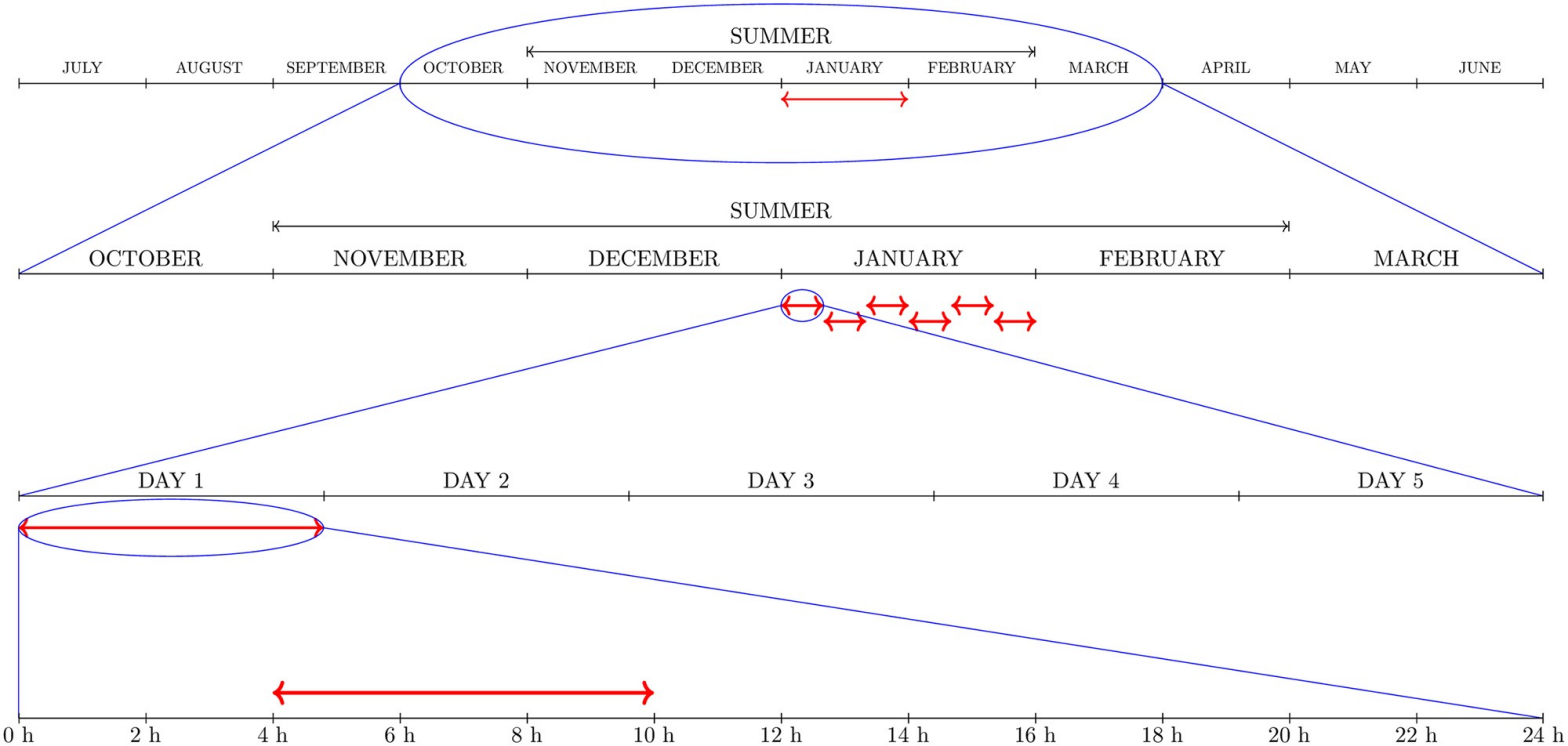
PUBL policy. Adulticide was applied every summer for a period of a hundred years. Six cycles of adulticide spraying were applied each year. Each cycle consisted of one daily application, which remained active without any decay for a quarter of a day, and a rest interval of 4 days.

**Fig 3:**
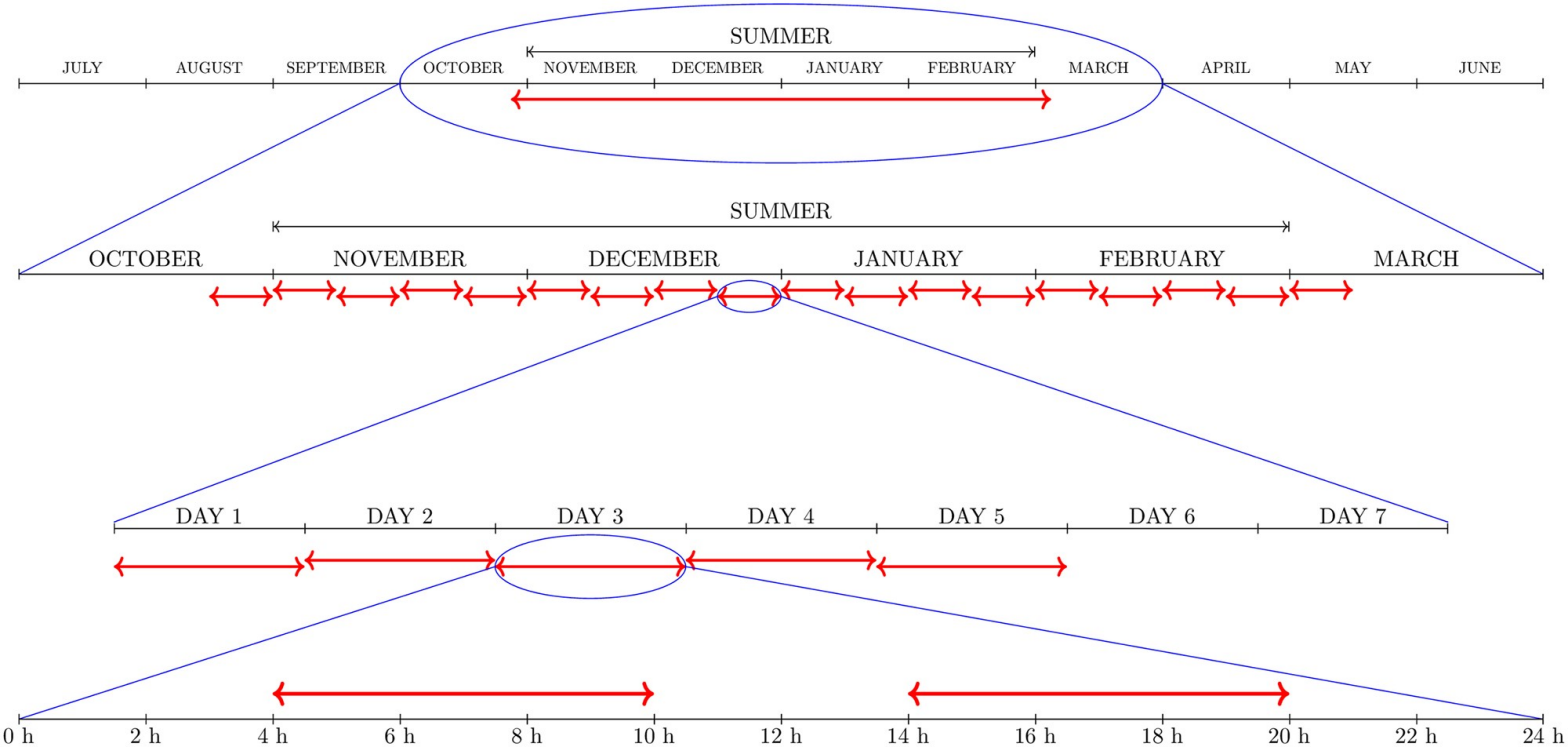
PRIV policy. Adulticide was applied every summer for a period of a hundred years. Eighteen cycles of adulticide spraying were applied each year. Each cycle consisted of two daily applications, that remained active without any decay for a quarter of a day, for 5 days and a rest interval of 2 days.

**Fig 4:**
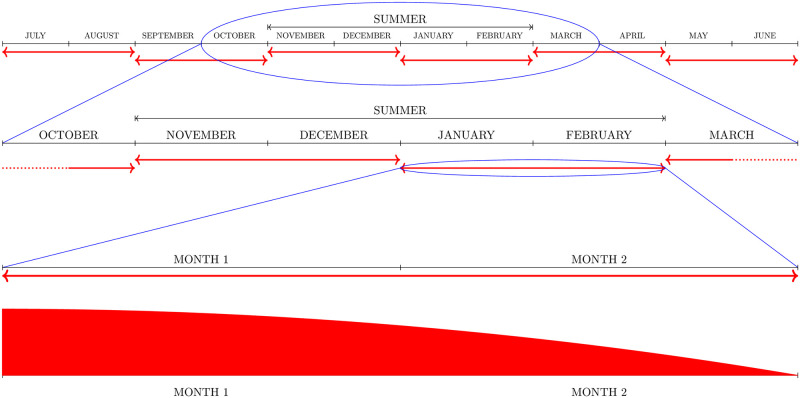
Larvicide policy. Larvicide was applied every two months for a period of a hundred years. Six cycles of larvicide dispersal were applied each year. Larvicide remained active for two months with decay that followed a parabolic function.

Their pictorial description together with further details of the PUBL application policy and of the most intensive application policy denoted as PRIV are given in Figs 2–4.

## Results

We now present a series of results that were obtained through in-silico experiments using the model given by Eq (1) together with the parameters described in Mathematical model. These experiments consisted of the application of insecticide using one of the two annual policies described before for a period of no less than one hundred years. The simulations were run for a selection of fitness costs and adulticide efficacy values. For the fitness costs, we considered values ranging from 0.005 to 0.15 in the set $C_{\text{values}}=\left\{0.005,0.01,0.05,0.1,0.15\right\}$. Whereas for the adulticide efficacy three different values were used within the set $K_{\text{values}}=\left\{1\%,6\%,10\%\right\}$. The median values—0.05 for the cost C, and 6% for the efficacy K—can be considered reasonable values for these parameters, even in the light of much uncertainty about their true values—see also the remarks in The populational control policy Discussion. In the experiments performed, resistance fixation was deemed to have occurred when the R allele prevalence frequency rose to values near 1—i.e. above 0.995. The initial prevalence frequencies were taken as 2% for the R allele and 98% for the S allele.

The primary intent of adulticide usage is a reduction on the total number of parous adult mosquitoes, thus reducing the biting nuisance and possibly diminishing the *R*_0_ for the associated arboviruses infections. Therefore, we also present the evolution of the parous mosquito population. This is done by plotting the annual average size of the parous population in the presence of insecticide control relative to that of an uncontrolled population—we will write annually averaged parous ratio for short.

### The PUBL policy

**Fig 5:**
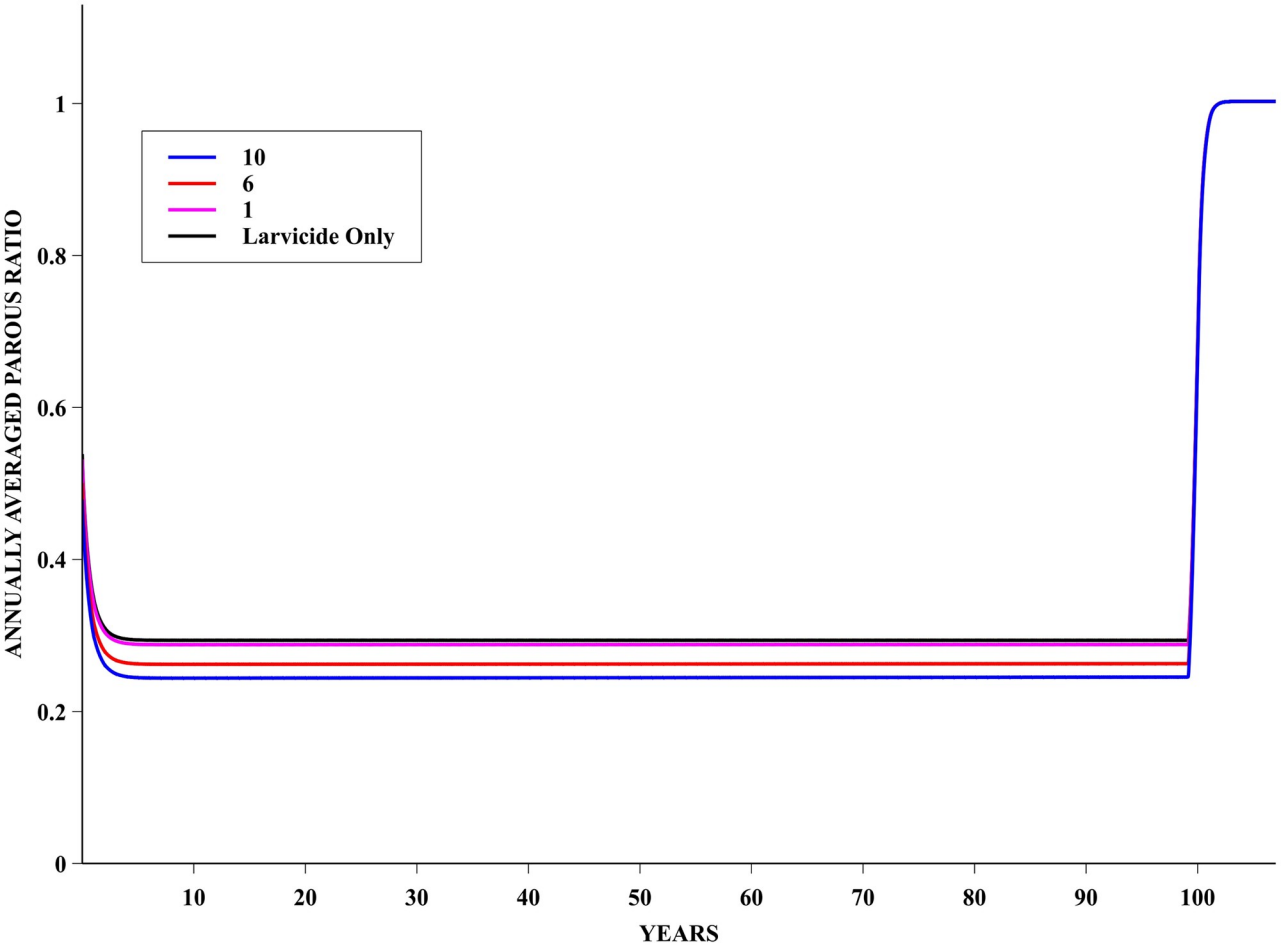
PUBL Policy—Annually averaged parous ratio dynamics. Evolution of the annually averaged parous ratio for a simulation following the PUBL insecticide application policy. Data with fitness cost of 0.005 and adulticide efficacy of 1%, 6% and 10%. For comparison sake, the evolution of this ratio for a population only subjected to larvicide (90% efficacy) is also shown.

The evolution of the parous population, under the PUBL policy applied for 100 years, is presented in Fig 5. It can be seen that a somewhat small further reduction on the average size of this population is achieved in comparison to the reduction obtained with the use of larvicide only—this additional reduction is larger the higher is the adulticide efficacy. As expected, the population reverts back to an essentially non-resistant one soon after the control is ended at 100 years.

**Fig 6:**
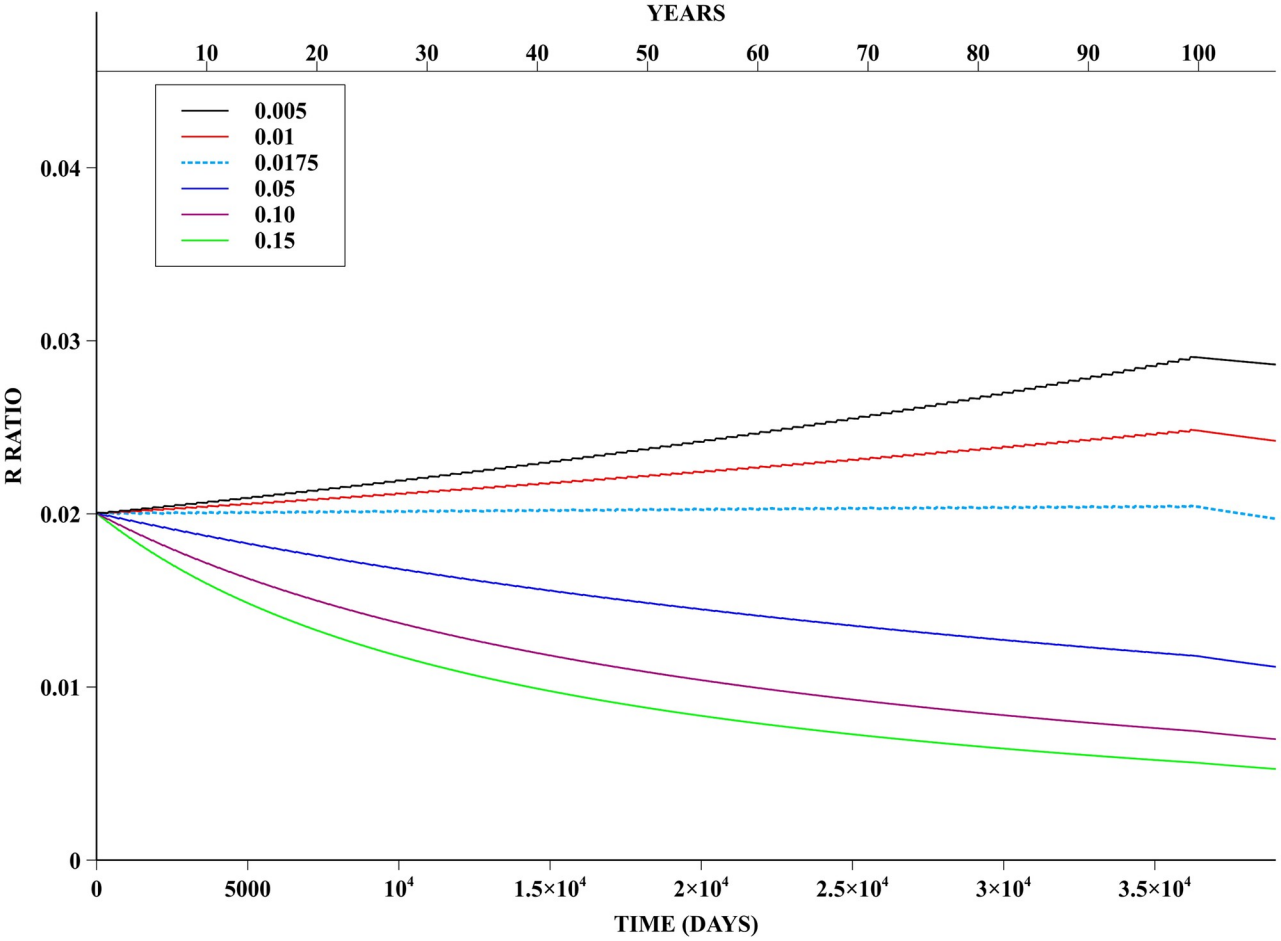
PUBL Policy—Adulticide efficacy of 10%. Prevalence frequency of R allele for simulations following the PUBL insecticide application policy. Data with fitness costs of 0.005, 0.01, 0.05, 0.10, 0.15 and adulticide efficacy of 10%. In addition, data for the neutral fitness cost of 0.0175 is also shown.

For the PUBL policy, ***NONE*** of the pairs of fitness cost and adulticide efficacy values yielded resistance fixation, as previously defined, over the considered timespan. Evolution of the R allele prevalence frequency under this PUBL policy for an adulticide efficacy of 10% can be seen in Fig 6. Even in this worst case scenario, i.e. fitness cost of 0.005 and adulticide efficacy of 10%, the prevalence frequency of the R allele never exceeded 3%. We point out, however, that for sufficiently small fitness costs the mutation is advantageous and then the R allele frequency increases in time—thus if we had applied the policy for a sufficiently long time we would observe the development of resistance. For the specific case of the PUBL policy and a 10% adulticide efficacy, we find that a fitness cost of 0.0175 balances the positive selection pressure of the insecticide application with a reasonable accuracy for the duration of the insecticide application of 100 years.

### The PRIV policy

**Fig 7:**
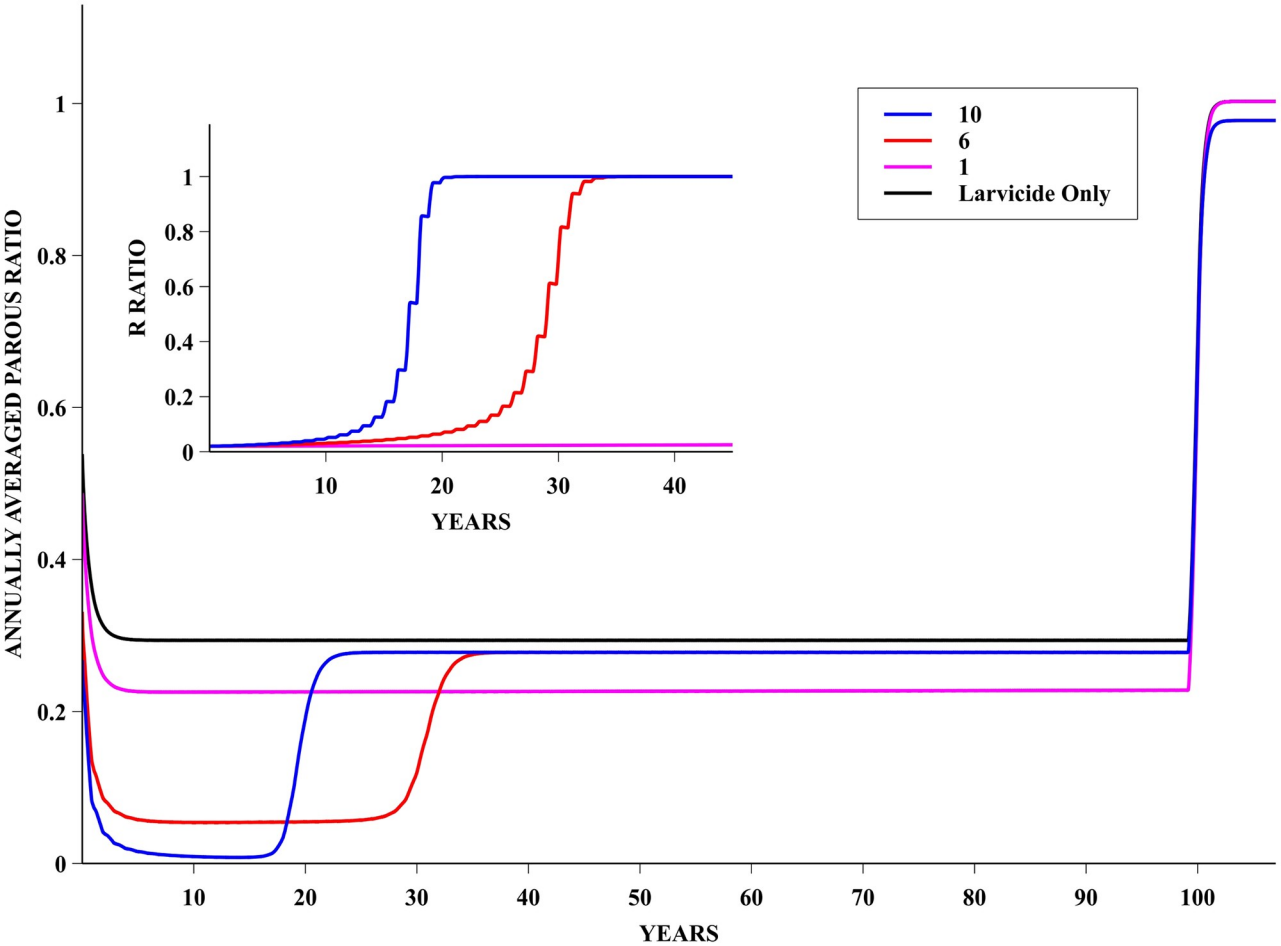
PRIV Policy—Annually averaged parous ratio dynamics. Evolution of the annually parous ratio for a simulation following the PRIV insecticide application policy. Data with fitness cost of 0.005 and adulticide efficacy of 1%, 6% and 10%. For comparison sake, the ratio for a population only subjected to larvicide (90% efficacy) is also shown. In the inset, the frequency of the allele R in the population for adulticide efficacies of 10% and 6% are plotted—the sharp rise of this frequency towards unity coincides with the also sharp reduction in the control efficacy.

The evolution of the parous mosquito population for the PRIV policy is presented in Fig 7—as with the PUBL policy the application was over 100 years. It can be seen that a drastic further reduction on the average number of parous mosquitoes is achieved, when applying adulticide with efficacies of 10% and 6%, in comparison to the reduction obtained with the use of larvicide only. However such a reduction lasts only until resistance builds up, which happens relatively fast, and then the average size of this population returns to about the same level as the one obtained with application of larvicide only. Similarly to the in-silico experiment with the PUBL policy, the population reverts back to an essentially non-resistant one soon after the control is ended at 100 years.

**Fig 8:**
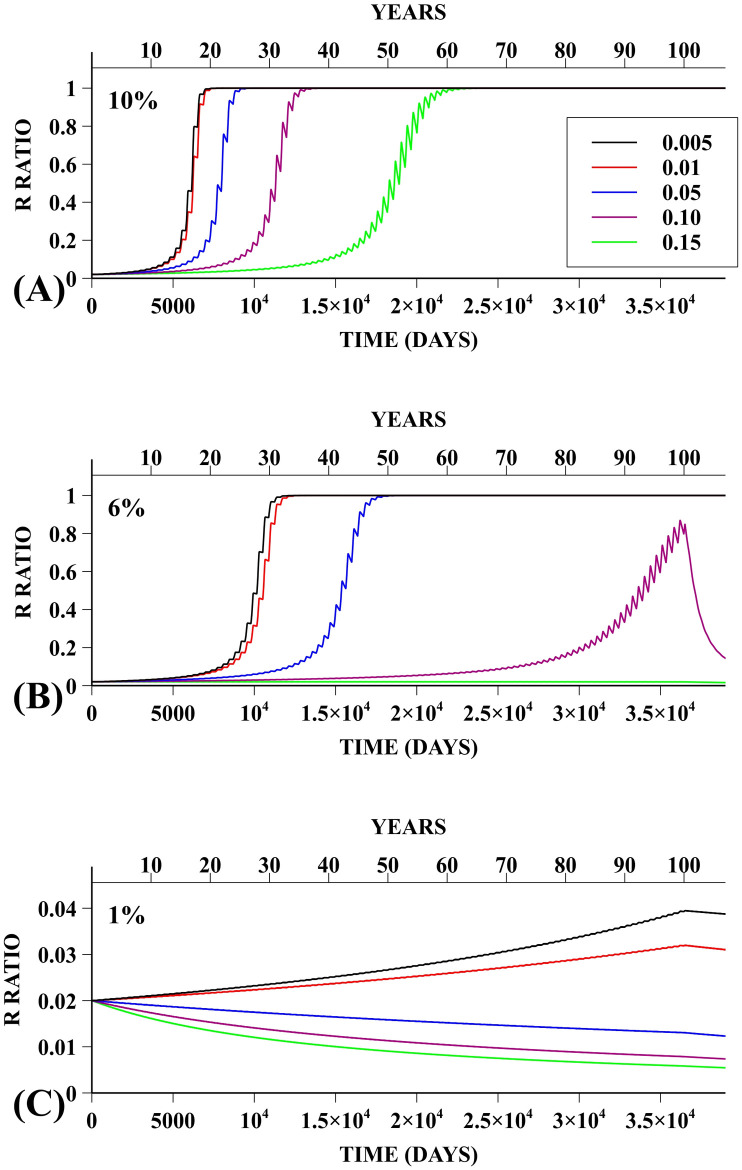
PRIV Policy—Adulticide efficacy of 10%, 6% and 1%. Prevalence frequency of R allele for simulations following the PRIV insecticide application policy. Each panel displays data with fitness costs of 0.005, 0.01, 0.05, 0.10, 0.15 and different adulticide efficacies: 10% **(A)**, 6% **(B)** and 1% **(C)**.

For the PRIV policy, resistance fixation, as defined above, was observed for several combinations of fitness costs and adulticide efficacies—see Table 2 for a summary of the results. For adulticide efficacy of 10%, the resistant allele was advantageous for all fitness costs considered. However, for adulticide efficacy of 6% fixation was not observed over the 100 years timespan for fitness costs of 0.1 and 0.15; in these cases fixation would occur after progressively larger timespans. For an efficacy of 1%, the results are qualitatively similar to the ones obtained under the PUBL policy with 10% efficacy—resistance is advantageous for sufficiently small fitness costs, but for the considered costs the growth over time is small and fixation would only be observed after a very long time. On the other hand, had we considered higher fitness costs, we would observe situations where resistance would be disadvantageous even for efficacies of 6% and 10%. Evolution of the R allele prevalence frequency under the PRIV policy for the various adulticide efficacies and fitness costs can be seen in Fig 8.

**Table 2:** Resistance development dependance on adulticide efficacy and fitness cost for the PRIV policy.

EFFICACY	1%	6%	10%
COST			
0.005	***—***	RESISTANCE	RESISTANCE
0.01	***—***	RESISTANCE	RESISTANCE
0.05	***—***	RESISTANCE	RESISTANCE
0.10	***—***	***—***	RESISTANCE
0.15	***—***	***—***	RESISTANCE

Interestingly, for the combinations of pairs of parameters values that yielded resistance fixation, a sigmoidal behaviour was observed with a sharp transition in the R allele prevalence frequency. This sharp transition period lasted for a very short time, i.e. less than 10 years (see Fig 8).

Finally, we want to point out that Table 2 suggests the existence of a boundary in the parameter plane given by efficacy and fitness cost, for a given control policy. Along this boundary, resistance is neutral, whereas it is dominating on one side and dominated on the other side. Notice, however, that while domination is a necessary condition for fixation, the timespan needed for it to occur may vary considerably.

## Discussion

This work is a first attempt to evaluate the development of A. aegypti insecticide resistance using a mathematical model for real world scenarios. As we shall discuss below, the use of such modelling approach might help shed light on some aspects of insecticide resistance development, as well as bring into attention unexpected features.

As in any modelling effort, the choice of model and parameter values might significantly change the outcome. In this vein, while the model adopted is likely to be oversimplified to be used quantitatively, previous experience [35, 38] with models in the same class suggests that it should be able to capture enough biological and ecological features.

Regarding the choice of parameters, all but three could be easily retrieved from the literature. The three remaining ones are the fitness costs associated with resistance and the efficacies of larvicide and adulticide. The literature on insecticide efficacy is not extensive, but it is usually agreed that larvicide are very efficient—cf. [34–36]. On the other hand, quantification of the efficacy of the adulticide has more widespread admissible intervals, and we used a more conservative parametrisation motivated by studies on insecticide in agricultural use [37]. The fitness cost associated to resistance is a controversial topic and we considered a wider range of values, while allowing for small values also as a worst case scenario analysis.

Our results show that, even for a long period of application such as 100 years, the insecticide policy prescribed by the Brazilian Ministry of Health did not lead to fixation of adulticide resistance. However, for intensive usage, such as the one described by the PRIV policy, resistance development occurred within a relatively short period.

It should be noted that, in the field, in the medium or long term, compensatory genes can also be selected in natural populations that decrease the fitness costs associated with insecticide resistance [30]. In these cases, the PUBL policy could result in higher R frequency levels than those here attained. Additionally, following the PRIV policy, selection of compensatory genes that reduce the costs of fitness, could induce fixation of insecticide resistance even in higher restrictive conditions than those here depicted—or faster than we found in our model.

Indeed, it has been noticed, in the real world, the very rapid increase in resistance levels, in periods that can be several times smaller than those shown here. Both compensatory genes and the seasonal intensification of insecticide use can contribute to this. An example is the uncontrolled domestic adulticides use during summer periods, or when new dengue outbreaks are disclosed [7–9, 20, 24, 25, 28, 32, 39].

Although the very intensive insecticide application policy PRIV might be considered unrealistic, it has been observed in loco in Brazilian high-middle class gated communities. Moreover, it serves as a case demonstration that excessive, very often unregulated, usage of insecticide leads to resistance development—for a thorough discussion on the evolutionary pressure by insecticide applications see [40]. The PRIV policy results suggest that this model is capable of explaining the development of insecticide resistance, as indicated by an increase in the frequency of specific genotypes, even in places where the particular chemical was not employed any longer by governmental agencies but was available off-the-shelf [9, 28, 39].

Interestingly, as can be observed in Fig 7, employment of an intensive adulticide policy leads to a short-lived reduction on the number of adults. It was also observed that, the higher the efficacy of the adulticide, the shorter the length of time of significant adult population reduction. This might be explained by the fact that the speed of resistance development correlates positively with adulticide efficacy. In other words, the more effective the adulticide, the faster the establishment of resistance, and the shorter the lifespan of the adult population reduction ascribed to the adulticide.

Moreover, for the PRIV application policy, there are adulticide efficacy and fitness cost combinations that promote a significant reduction on the number of adults below the reduction that can be ascribed to larvicide only. Therefore, possibly reducing the *R*_0_ of associated arboviruses infections to values below the epidemic threshold. However, this beneficial effect is short lived as the frequency of the allele R present in the population increased significantly, leading to resistance fixation. In other words, such a strategy might buy comfort for some time but it will, in the medium term, render the adulticide control ineffective.

It might be argued that by assessing the frequency of the allele R in the population in field experiments, it would be possible to promote alternation of the chemicals used as adulticide. However, field experiments to assess genetic frequencies are based on capturing and analysing only few specimens, therefore the inherent error in the estimated frequency is quite high. In addition, recessive genetic traits spread unnoticed in the populations, through heterozygous individuals, as evidenced by the long lasting allele R prevalence frequency in Figs 6 and 8—see [38]. Therefore, these traits can only be identified when their frequency is high enough to allow the appearance of detectable homozygotes. However, from then on, resistance may increase extremely rapidly. This is known as tipping point as noted in this WHO report [41].

Under-estimation of the resistant population fraction, combined with the time required for governmental agencies to implement changes, might cause resistance development for all the chemicals involved in a rotation scheme. For Brazil the time required for implementing changes for insecticide rotation is at least two years. The consequence of this rotation scheme failure might be even harsher as the inventory of existing chemicals is fairly finite and it is quite time consuming and pretty expensive to develop new ones.

The picture is even more complex, as many insecticides are currently easily and freely found in the retail market for domestic and private use, which is a manifestation of the protagonism attributed to chemical control, and especially to the chemical control of adults, when indeed it is vector control that is on the agenda. In fact, there is a noticeable lack of regulation for the marketing of insecticides, a situation that should be revised, as it contributes significantly to the early loss of the few control alternatives available for public health.

From a theoretical perspective, our results suggest the existence of a neutral boundary in the efficacy–fitness cost parameter space. The geometry of this boundary will certainly depend on the policy adopted. However, crossing this neutral boundary is not sufficient for fixation of the resistant allele to occur within a relevant timespan. Indeed, although the growth rate increases as one gets far into the region where resistance is dominating, the dependence of fixation time on this path is not straightforward and additional investigation is needed.

We expect the present model to be useful as a starting point to studies providing further insight on qualitative differences of control strategies. Indeed, these studies should comprise different strategies of vector control, which must include mechanical control such as removal of eggs and larvae, as well as the impact of those insecticide application policies in terms of the elapsed time for resistance fixation. For these, further analysis of insecticides characteristics and data on resistance and fitness evolution are needed so that the model can be confronted with field data.

Along this line, the authors are currently investigating the existence of possible strategies that might be more efficient than the PUBL policy, but which would do it so without diminishing significantly the length of time until adulticide resistance development.

## Supporting information

S1 Text Supplementary information. Additional details regarding the initial condition used and the computational implementation.
(PDF)
