# Supplementary material for: From resistance to persistence: Insights of a mathematical model on the indiscriminate use of insecticide
Source: PLoS Negl Trop Dis. 2020 Nov 18;14(11):e0008862. doi: 10.1371/journal.pntd.0008862 (PMC7723293; doi:10.1371/journal.pntd.0008862)
Supplement: S1 Text — Additional details regarding the initial condition used and the computational implementation. (PDF) [file pntd.0008862.s001.pdf]

From Resistance to Persistence: Insights of a Mathematical Model on the Indiscriminate Use of Insecticide

Helio Schechtman, Denise Valle, Max Souza

Supporting Information

1 Initial conditions

The initial condition for the simulation is obtained by redistributing a purely susceptible population — see the SI in [1] — into the three genotypes. This distribution is done according to the Hardy-Weinberg proportions of 2% for the R allele and 98% for the S allele to generate the mainly susceptible population described in the main text.

Stage	SS	SR	RR
EGG	469300000*0.9604	469300000*0.0392	469300000*0.0004
LARVA	2101800*0.9604	2101800*0.0392	2101800*0.0004
PUPA	343790*0.9604	343790*0.0392	343790*0.0004
NON-PAROUS	934610*0.9604	934610*0.0392	934610*0.0004
PAROUS	2823400*0.9604	2823400*0.0392	2823400.*0.0004

Table 1. Distribution of a mainly susceptible population

Computational implementation

The model described above was implemented in Octave version 4.2.2 using the Lsode routine for integration.

References

1. Schechtman H, Souza MO. Costly Inheritance and the Persistence of Insecticide Resistance in Aedes aegypti Populations. PLoS ONE. 2015;10(5):e0123961. doi:10.1371/journal.pone.0123961.
